# Bioactivity of Isoflavones: Assessment through a Theoretical Model as a Way to Obtain a “Theoretical Efficacy Related to Estradiol (TERE)”

**DOI:** 10.3390/ijms11020480

**Published:** 2010-02-02

**Authors:** Maria da Graça R. Campos, Miguel Pires Matos

**Affiliations:** 1 Center of Pharmaceutical Studies, Laboratory of Pharmacognosy, Faculty of Pharmacy, University of Coimbra, Polo III, Azinhaga de Santa Comba, Coimbra, 3000-548, Portugal; 2 Bluepharma, Industria Farmaceutica, S. A. Rua da Bayer, São Martinho do Bispo, 3045-016 Coimbra, Portugal; E-Mail: mmatos@bluepharma.pt

**Keywords:** soy extracts, isoflavones, oestrogen-like, menopause, TERE

## Abstract

The increase of human life span will have profound implications in Public Health in decades to come. By 2030, there will be an estimated 1.2 billion women in post-menopause. Hormone Replacement Therapy with synthetic hormones is still full of risks and according to the latest developments, should be used for the shortest time possible. Searching for alternative drugs is inevitable in this scenario and science must provide physicians with other substances that can be used to treat the same symptoms with less side effects. Systematic research carried out on this field of study is focusing now on isoflavones but the randomised controlled trials and reviews of meta-analysis concerning post-menopause therapy, that could have an important impact on human health, are very controversial. The aim of the present work was to establish a theoretical calculation suitable for use as a way to estimate the “Theoretical Efficacy (TE)” of a mixture with different bioactive compounds as a way to obtain a “Theoretical Efficacy Related to Estradiol (TERE)”. The theoretical calculation that we propose in this paper integrates different knowledge about this subject and sets methodological boundaries that can be used to analyse already published data. The outcome should set some consensus for new clinical trials using isoflavones (isolated or included in mixtures) that will be evaluated to assess their therapeutically activity. This theoretical method for evaluation of a possible efficacy could probably also be applied to other herbal drug extracts when a synergistic or contradictory bio-effect is not verified. In this way, it we may contribute to enlighten and to the development of new therapeutic approaches.

## 1. Introduction

Since in 1984 Setchel *et al*. [1] discovered in human urine the compound equol, one of the metabolites of daidzin only produced *in vivo* by 30–50% of the population, many epidemiological studies began to correlate the lower observed incidences of hormone-dependent tumours in the East Asian population with the consumption of soy and soy derived foods. Since then, many researchers, all over the world turned their attention to isoflavones and initiated studies to explore its effects. Today, one of the major concerns about isoflavones lays in the definition of a suitable dose-dependent effect, thereby creating new therapeutic options.

Nowadays, over the counter (OTC) tablet preparations and nutraceuticals with isoflavones extracted from soy and other plants are widespread around the world. These OTC medicines are often used for postmenopausal treatment, like Hormone Replacement Therapy (HRT) with oestrogen and/or progesterone (or bio-identical hormone therapy, which means a medication that, provides one or more of these hormones as the “active ingredient”-www.menopause.org/bioidentical.html).

The ability that isoflavones have to mimic oestrogen molecules in their capacity to bind to estrogens receptors (ERs) is most evident in genistein and daidzein and their derivatives. The characteristic way that isoflavones bind to oestrogen receptors (ERs), mainly to beta-receptors (ERs-β), makes them promising molecules in replacing hormones for therapeutic purposes.

Very early, in the beginning of the research in this field of study, it became evident that isoflavones were less potent than endogenous and synthetic hormones but it also became obvious that they had fewer side effects. Despite the huge amount of results from many epidemiological trials, it is not possible to make a good correlation between isoflavones and soy extracts being sold today [2–5].

Epidemiological data prove that both genders of Asian population are less affected by hormone-dependent tumours. This fact as been directly related to the cultural diet rich in soy, but other factors could be also involved, such as the low amount of saturated fatty acids ingested, different ways to deal with stress and possible low levels of cortisol, among others, since Indians did not eat much soy and are also protect against these tumours.

Since the most active isoflavone, which is also the main isoflavone found in soy seeds, is genistein and its derivatives, we decided to compare OTC tablets containing isoflavones extracted from soy, available in the Portuguese market and the isoflavones extracted from soy seeds supplied by the United States Department of Agriculture (USDA) Germplasm bank. These formulations where all indicated for the treatment of hot-flushes and post-menopausal related problems where the content in isoflavones it was critical for the referred bioactivity. The major conclusion of this comparative study was that important differences existed between the OTC formulations and the content of genistein and other isoflavones in raw soy seeds extracts. The quantification of the isoflavones present in OTC formulations was sometimes lower than the expected levels (the isoflavones content was analysed by HPLC/DAD and LC/MS-MS) [6–8]. These differences were extendable in many cases to the kind of isoflavones and in other cases to the lack of isoflavones themselves.

If we consider that it is not yet clear how much of a dose of isoflavones is necessary to observe a physiological effect and if the soy protein has any synergic contribution, we should be concerned about what is really contained in the OTC Tablet preparations of soy isoflavones and what kind of quality control should be established [7–9]. The relationship between the structure and the bioactivity has been evaluated by different authors and is well established for the main aglycones like genistein, daidzein, glycitein, biochanin-A and formononetin [10,11].

Despite the number of clinical trials performed with isolated isoflavones or with isoflavones included in soy or red clover food products, results did not clarify its efficacy or whether they could be used safely as a treatment as it is claimed. On the other hand, the U.S. Food and Drug Administration (FDA) back in 1999 made a statement that soy protein can be used with beneficial results in the prevention of cardiovascular diseases, and recommended that its extracts should be standardised, however the amount of isoflavones that can be on the product was not defined.

The OTC tablet preparations of isoflavones extracted from soy that we analysed showed as major bioactive compounds the glycosylated isoflavones genistin, daidzin and glycitin and minor amounts of the malonyl and/or methylated derivatives of genistin and daidzin. The aglycones only appeared in very low amounts. The seeds, supplied from the USDA Germplasm Bank, have a mixture of most of these compounds (conjugated or not) but also have low amounts, some times only traces, of the aglycones [6–9].

The evaluation of the efficacy of several extracts and the different results of clinical trials carried out with soy extracts or isolated isoflavones turns its usage into a very controversial subject. Scientists are now aware of the phenomena. Many are now studying the effects of formulations available to post-menopausal women. Given the difficulty of prescribing such formulations, the aim of the present work was to establish a theoretical calculation suitable to be employed as a way to estimate the “Theoretical Efficacy (TE)” of a mixture of different bioactive compounds in a way to obtain a “Theoretical Efficacy Related to Estradiol (TERE)”. Ultimately, different extracts could be compared, even when the relative amounts present in the extracts are very different.

## 2. Experimental Setup

Data from literature and reviews of selected clinical trials: a literature survey was made, using the following search engines: Elsevier-Science Direct, PubMed, SpringerLink, Taylor & Francis, Web of Science (ISI) and B-on. The search included the following keywords: *hot-flush, menopause, isoflavones, genist-, daidz-, glycit-, clinical trials*. We also used the selected papers by a panel of Phytohealth members that had reviewed studies and classified them according to the grading criteria proposed by Harbour and Miller [13] and the papers that Cassidy and colleagues [12] presented in 2006 in a literature search (up to June 2005) using MEDLINE (PubMed), EMBASE and the Cochrane Collaboration (The Cochrane Library). The search included the following keywords: *phytoestrogens, isoflavones, genistein, daidzein, equol, soy* (a); cross-referenced with the keywords: *post-menopausal, hot-flushes, osteoporosis, bone mineral density, bone metabolism, cardiovascular, endothelial function, vascular reactivity, blood pressure, lipid profile, breast, colon, cognition*. In order to make cross comparisons between studies that used a range of sources of isoflavones the doses of isoflavones were calculated as aglycone equivalents.

In this paper we use from the clinical data two important keys to the process; on one hand the relative proportions of the different isoflavones intake and on the other, the efficacy of the treatment in reducing hot-flushes that is one of the parameters more cited in the most of the essays. To do the theoretical calculation [14] these amounts of isoflavones were multiplied by the “ERs binding affinities” based on the values obtained in literature [10]. After applying the proposed model, “Theoretical Efficiency Related to Estradiol (TERE)” for each mixture of isoflavones, the “Theoretical Efficiency (TE)”, which can be used to compare the potential bioactivity, was also calculated.

## 3. Results and Discussion

### 3.1. Findings and Interpretation

The isoflavone content (daidzin and genistin and their derivatives) clearly differs between seeds and OTC tablet preparations extracted from soy. In these OTCs the main compounds are the glycosidic forms, glycitin, daidzin and genistin, but the relative proportions change from one sample to another. From the clinical trials used in the selected reviews, the levels of active compounds used to perform the assays differed significantly and the results are very difficult to compare.

We found in literature that there is a consensus that glycosylated and methylated isoflavones have an entero-hepatic absorption where they undergo hydrolysis and afterwards are converted to the sulphonated or glucuronated forms. The aglycones undergo the same metabolization.

Daidzein and genistein do not have the same binding affinity to alpha- and/or beta-ERs [10]. Also known and frequently mentioned by several authors is that the linkage of oestrogens or oestrogenic compounds to alpha-ERs could be dangerous with breast cancers because it could aid the proliferation of damaged DNA in tumours [15,16]. In this paper we calculated the amounts theoretical linked from the different isoflavones to the alpha- oestrogen receptors as an add benefit (when added to the beta-receptors affinity) but also as a potential risk to be evaluated.

However, it is also accepted that beta-ERs are mainly located in bones, brain, thymus, bladder, cardiovascular system and its activation by estrogenic compounds, or compounds with the ability to mimic estrogenic molecules, such as phytoestrogens, can improve and prevent conditions like osteoporosis and cardiovascular diseases [17,18] and this will be used as the benefit parameter.

In global terms the total efficacy of the TERE will be determined adding these two values. However the amount linked to the alpha-receptors will be consider as a possible risk limitation that needs to be evaluated when the increased dose will induce an improved TERE but the risk assessment for the toxicity can be a handicap.

In order to allow an assessment between results obtained from clinical trials and different substrates submitted for therapeutic evaluation, the following calculation is proposed.

To demonstrate the calculation method, let us consider for example two different clinical trials, both using test formulas containing 100 mg of total isoflavones. In one of them the patients are taking pills with 25 and 75 mg of daidzein and genistein equivalents, respectively, but in the other trial patients ingest 75 mg of daidzein and 25 mg of genistein, which is exactly the inverse of the previous formulation. Looking at Table 1, it is possible to have an idea of the differences in affinity of isoflavones for alpha and beta-ERs, a fact that is well documented. It should also be remembered that bioactivity of estrogens, for example estradiol, is considered to be 100/100 to ERs, nevertheless, estradiol affinity is still slightly greater to alpha-ERs than to beta-ERs. In this paper we used the Fokialakis *et al.*, ERs affinity binding for estradiol for some isoflavones but the some theoretical calculations can be done with other affinities published. Utilising these TERE calculations will be possible to compare results of the bioactivity between various clinical trials or other kind of bio-evaluations.

Keeping the affinity differences in mind, the ER binding values for daidzein and genistein are a starting point to make the theoretical calculation of the efficacy of both mixtures (see Table 2). The overall values would be 33.5 and 11.2 of theoretical efficacy (TE), which is the same as saying that the TERE (theoretical efficacy related to estradiol) will be 1/3 (100% oestrogen binding/33.5 = 2.98 ≃ 3) and 1/9 (100% oestrogen binding/11.2 = 8.93 ≃ 9), respectively. The real efficacy of the second situation described maybe gives three times less activity that will be expected for the first mixture under evaluation. In fact, in the two example trials the efficacy is so different that probably in trial 1 we would find therapeutic bioactivity but in trial 2 we could probably have a very lower effect to obtain relevant results.

This is currently the case of the clinical trials conducted with botanical extracts if they are not well characterized and/or not comparable in relation to the relatively amounts of the bioactive compounds. It is very common to see reported in literature that in a given trial was used an extract with bio-compounds without saying exactly which molecules were in the extract.

Since isoflavones and other molecules are able to mimic estrogens and they all have a different affinity for the receptors involved in the therapeutic activity, the results cannot be compared. For example, opium has two main alkaloids, morphine and codeine, both with analgesic activity but the first is much more active that the methylated derivative (codeine). The therapeutic efficacy of different mixtures of an opium extract would be therefore dependent on the relative amounts of each compound in them even when calculated with these TE calculations.

Applying the same methodology as in Table 2 for two more examples, the results show the value of this approach (Table 3). The efficacy is different depending on mixture composition and this should therefore provide a better understanding of the results obtained in different trials (Table 4).

For all of them, nevertheless, the risk-benefit relationship is determined as roughly 2% and 98% respectively. The increase of dosage can signify an increase of the amount that can be related to the risk, *i.e.*, molecules that have a preferential binding to alpha-ERs.

Once the maximum binding rate of alpha-ERs is determined, the risk of possible tumour cell proliferation by the estrogenic activity of these compounds will be lower and the safety of these OTC tablet preparations of isoflavones extracted from soy, red clover, etc, will be more predictable. This is an example of the application for the TERE.

For example, in the first calculations (Table 2) the determined risk is 1.9 and 2.1% (trial 1 and trial 2) but the amounts of compounds capable to be bound to alpha-ERs are different. In the second trial 0.24 could be under the dose that might induce proliferation of human breast tumour cells and 0.65 (first trial) could be related to a high risk intake.

Highly purified isoflavones, soy foods and soy supplements may stimulate the growth of pre-existing oestrogen-dependent breast cancer tumours. Using an animal model, Ju and colleagues [19] compared different soy foods and supplements containing the same concentrations of genistein and found that dietary soy products that contain isoflavones in more purified forms were associated with greater tumour growth. This is one of the most important issues related to the safety of these products that needs to be clarified as soon as possible.

The *in vivo* potential of equol and flavonoids, such as genistein, equol, apigenin and kaempferol was investigated by Breinholt and colleagues [20], in immature female mice. They verified that genistein and equol, administered by gavages for four consecutive days [post-natal day (PND) 17–20, 100 mg/kg body weight], was found to significantly increase uterine weights and the overall uterine concentration of alpha-ERs.

In kaempferol- and equol-exposed mice the cytosolic alpha-ERs concentration was significantly increased when compared to the solvent control, which is speculated to result in an increased sensitivity of the uterus to subsequently encountered estrogens. Oral administration of equol, genistein, biochanin-A and daidzein to 6-week-old female mice revealed a large variation in their systemic bioavailability. Other researchers found similar data. Bioavailability, metabolism, the ability to alter alpha-ERs distribution in the uterus and the estrogenic potential of parent compounds and metabolites may consequently contribute to the differences in “*in vivo*” estrogenicity of dietary flavonoids.

The essays published so far did not reveal yet the full risk assessment of these concentrated extracts. A study reported in the May 2004 online issue of the journal *Carcinogenesis*, suggests that isoflavone-containing products consumed in the US may have lost many of the biologically active components in soy and, therefore may not have the same health benefits as traditional soy foods.

In fact, the profile of the isoflavones is different in soy seeds [9] and in the OTC or in the nutraceuticals made with soy extracts and available in the market, for example in Portugal [8]. This fact implies the need to introduce an extra evaluation for the theoretical evaluation of the bioactivity which additionally can be a tool to add researchers to compare all these variable mixtures.

This TERE can be supported by the data collected by Williamson-Hughes *et al*. [35] and by Kurzer, 2008 [36]. They found that the essays carried out with 10 mg of the genistein the data were not relevant. Using our calculation, TERE will be 1/22.8, and the low level for genistein that’s induce a significant bioactivity improving a decrease of the hot-flushes needs to be at least 15 mg. Appling again the calculation, TERE will be 1/15.2. Briefly to just comparing these two results we could say that under a TERE equal or under 1/15 the probability to have an important bioactivity is too low and above this value is possible to achieve relevant results, what confirms that our purpose can be used as a start point for the evaluation of the extracts chosen for clinical trials.

## 4. Conclusions

Much research as been published relating to the issue of “phytoestrogens in human health”, but few recommendations can be made because most of the results seem to be controversial [12]. Maybe, in the future, using the theoretical calculation method outlined here it will be possible to better compare results from trials worldwide. This methodology can give an indication of the therapeutic effects that can be expected, but not in cases where a synergistic or antagonist effect is present.

The authors declare that they are not for or against the use of isoflavones in human health applications and that they just wish to attempt to clarify this controversial issue. For instance, the verified estrogenic bioactivity shows an important relationship between genistein dose and the reduction of hot flushes and night sweats. These unpleasant effects observed in post-menopausal women are caused by lower oestrogen levels that fail to regulate body temperature via the hypothalamus. The anti-dopaminergic effect induced by estrogens [21] can be replicated by genistein in women using isoflavone supplements thus inducing a better regulation of body temperature in postmenopausal women. However, the possible side effects of this administration are apparently not yet evaluated.

In order to prevent the risks that this therapeutic option can cause in long term use, much research still needs to be done to prove the safety of isoflavones. Fortunately, many of the issues commented here will be under investigation. Studies of the effects of soy isoflavones found in dietary supplements on various parts of the body are under way in various research groups.

Some of them are investigating how different doses of isoflavones and the timing of exposure affect breast, brain and adipose tissue, and how the interaction between isoflavones and oestrogen receptors takes place. More information is needed on the biological effects of pure isoflavones and complex mixtures of the various soy isoflavones commonly found in commercial OTC tablet preparations of isoflavones extracted from soy and if the matrix induces changes in the bioability of these compounds.

## Figures and Tables

**Table 1.** Example of ERs affinity binding for estradiol and some isoflavones [10].

**Compound**	**Alpha-ERs**	**Beta-ERs**
estradiol	100	≤100
daidzein	0.031	0.020
genistein	0.860	43.9
formononetin	0.084	0.017
biochanin A	0.094	0.010

**Table 2.** Theoretical calculation of the efficacy of two mixes of isoflavones using the ER affinity binding values for the compounds included daidzein and genistein.

	***Assay-1***	Receptor Type		Receptor Type		***Assay-2***
	Intake (mg/day)	Alpha-ERs	Beta-ERs	Alpha-ERs	Beta-ERs	Intake (mg/day)
daidzein	25	0.008	0.005	0.023	0.015	75
genistein	75	0.645	32.9	0.215	10.97	25
Total	100	0.653	32.905	0.238	10.985	100
**TE**	33.5 * = (0.653 + 32.905)			(0.238 + 10.985) = 11.2*		
**TERE**	1/3* of the theoretical activity of the estradiol			1/9* of the theoretical activity of the estradiol		
RISK		**1.9%**		**2.1%**		
BENEFIT			**98.1%**		**97.9%**	

* Theoretical values without units

**Table 3.** Two more examples of different clinical trials. **(a)** ***Clinical trial–1.*** OTC preparation of isoflavones extracted from soy ***A*** with 60 mg daily intake - 75% of genistein and 20 % of daidzein and 2.1% of glycitein / results obtained: reduction of about 50% of hot flushes in the population involved in the test [22]. To do the calculation for glycitein we used the same affinity where that we have for daidzein as the amount was so low and the transformation in gut will result theoretically in the same metabolites and the contribution to the bioactivity is almost irrelevant. **(b)** ***Clinical trial–2***. Performed with isoflavones extracted from red clover. The endpoint was the effect on bone density. Non-significant results were obtained [23]. **(c)** ***Clinical trial–3.*** Performed with soy germ to determine its effect on bone density. The results suggest attenuation on bone loss [24].

**(a)**			
**Soy Isoflavones**	Intake (mg/daily)	Alpha-ERs	Beta-ERs
daidzein	12	0.00372	0.0024
Glycitein*	1.26	0.000492	0.000252
genistein	45	0.387	19.755
total	60	0.39165	19.758
**TE**	20.148*		
**TERE**	1/5* (4.98 ≃ 5)		
**(b)**			
**Red Clover Isoflavones**	Intake(mg/daily)	Alpha-ERs	Beta-ERs
daidzein	0.1	0.000031	0.00002
formononetin	16	0.01344	0.00272
genistein	1	0.0086	0.439
biochanin A	26	0.02444	0.0026
Total	43.5	0.283511	0.44434
**TE**	0.727*		
**TERE**	1/137.4*		
**(c)**			
**60g Soy Germ**	Intake (mg/daily)	Alpha-ERs	Beta-ERs
Daizein + glycitein	1206	0.374	0.241
genistein	185	1.591	81.215
total	1392	1.965	81.456
**TE**	83.421*		
**TERE**	1/1.2*		

* Theoretical values without units

**Table 4.** Comparison of the TERE of different mixtures utilised in various clinical trials and the respective bio-effects observed.

**Clinical Trial reference**	**Results**	**Daily Intake**	**TERE**	**Risk Alpha-ERs**
[25]	↓ 24% hot flushes	50 mg genistein	1/4.47	0.43
[26]	↑ 3% BMD ↑ bone formation ↓ bone resorption	54 mg genistein	1/4.14	0.46
[27,28]	↑ nitrites or nitrates ↑ brachial artery FMD ↓ endothelin-1	54 mg genistein	1/4.14	0.46
[29]	↑ FMD	48 mg genistein 24 mg daidzein 8 mg glycitein	1/4.65	0.43
[30]	No change in FMD ↑ endothelin-independent vasodilation	40.9 mg genistein 40.9 mg daidzein 8.2 mg glycitein	1/5.46	0.37
[31]	No change in FMD	80 mg biochanin A	1/1201.92	0.075
[31]	No change in FMD	80 mg formononetin	1/1237.6	0.067
[32]	↑ arterial compliance	4mg genistein 3.5 mg daidzein 24.5 mg biochanin A 8 mg formononetin	1/54.77	0.065
[33]	↑ arterial compliance No change in FMD	76 mg genistein 37 mg daidzein 5 mg glycitein	1/2.94	0.67

FMD–flow- mediated dilatation BMD–bone mineral density.
